# Association Between Meal Regularity and Obesity Among Korean Older Adults: A Population-Based Study Using KNHANES VIII

**DOI:** 10.3390/healthcare14193278

**Published:** 2026-10-02

**Authors:** Hyo-Eun Park, Kyoung-Eun Jeong, Hye-Young Song

**Affiliations:** 1 Department of Nursing, School of Health Science, Hanseo University, 46 Hanseo 1-ro, Seosan-si 31962, Republic of Korea; lisa8413@gmail.com; 2 College of Nursing, CHA University, 120 Haeryong-ro, Pocheon 11160, Republic of Korea; poo607@naver.com; 3 Department of Nursing, Woosuk University, Samrye-eup, Wanju-gun 55338, Republic of Korea

**Keywords:** older adults, obesity, abdominal obesity, meal regularity, Andersen’s Behavioral Model

## Abstract

**Highlights:**

**What are the main findings?**

- Regular consumption of all three meal types was associated with 39% lower odds of general obesity and 34% lower odds of abdominal obesity among Korean older adults.
- Regular consumption of all three meal types was associated with lower odds of general obesity, while regular lunch consumption was also associated with lower odds of abdominal obesity.

**What are the implications of the main findings?**

- Meal regularity may be a behavioral factor warranting further evaluation in relation to obesity outcomes and healthy aging among older adults.
- Community-based meal programs, including congregate dining and meal-delivery services, may be considered potential strategies for future evaluation; their effectiveness was not examined in this study.

**Abstract:**

**Background:** Obesity in older adults is a major public health concern associated with chronic disease and reduced healthy life expectancy. This study examined the associations between habitual meal regularity and general and abdominal obesity among Korean older adults using Andersen’s Behavioral Model. **Methods:** Data from 3559 adults aged ≥65 years who participated in the eighth Korea National Health and Nutrition Examination Survey (2019–2021) were analyzed. Dietary behaviors included regular breakfast, lunch, and dinner consumption and the number of meal types regularly consumed. General obesity was defined as body mass index ≥ 25 kg/m^2^, and abdominal obesity as waist circumference ≥ 90 cm in men and ≥85 cm in women. Complex-sample hierarchical logistic regression analyses were conducted after adjusting for predisposing, enabling, and need factors. **Results:** Participants who regularly consumed all three meal types had lower body mass index and lower prevalence of general and abdominal obesity. In fully adjusted models, regular breakfast (adjusted odds ratio [aOR] = 0.64), dinner consumption (aOR = 0.51), and regular consumption of all three meal types (aOR = 0.61) were associated with lower odds of general obesity. Regular lunch consumption (aOR = 0.74) showed a nominally significant association with general obesity, but this association did not remain significant after the post hoc false discovery rate adjustment. Regular lunch consumption (aOR = 0.67) and regular consumption of all three meal types (aOR = 0.66) were also associated with lower odds of abdominal obesity. **Conclusions:** Regular meal consumption, particularly regular consumption of all three meal types, was associated with lower odds of general and abdominal obesity among Korean older adults. Meal regularity warrants further evaluation as a potential target in longitudinal and intervention studies, although causal relationships cannot be inferred from this cross-sectional study.

## 1. Background

### Research Necessity

Due to rapid economic growth and advances in medical technology, the proportion of older adults has increased substantially. Individuals aged 65 years or older comprised 12.2% of the population in 2013, are projected to account for 20% by 2025, and are estimated to reach 40% of the total population by 2050 [1]. This demographic trend reflects not only a transformation in population composition but also marks the transition to a super-aged society that will reshape healthcare, economic, and welfare systems.

Obesity among older adults has become a significant public health concern. In this population, obesity is more than a body composition issue; it serves as a substantial risk factor for premature mortality by contributing to serious chronic conditions, including hypertension, diabetes, and cardiovascular disease [2,3]. Age-related loss of muscle mass and increased visceral fat further exacerbate these issues, thereby reducing healthy life expectancy [4]. Although obesity has been recognized as a disease by the World Health Organization (WHO) [5,6,7], the prevalence of obesity in Koreans aged 70 years and older has increased consistently, from 29.5% in 2001 to 34.6% in 2023 [1]. This ongoing trend highlights the increasing socioeconomic burden associated with obesity amid a rapidly aging population.

From a biological perspective, older adults are predisposed to obesity because of age-related reductions in muscle mass and lower basal metabolic rates, even when caloric intake does not increase [8,9,10]. In addition, factors such as living alone, lower socioeconomic status, and decreased physical activity complicate the maintenance of healthy dietary patterns [11,12,13]. These factors highlight the multifaceted nature of obesity among older adults and the need to consider individual, socioeconomic, and behavioral characteristics when examining obesity-related factors.

Dietary habits, among various lifestyle-related factors, have been recognized as potentially modifiable factors associated with health outcomes [14,15,16]. Recent epidemiological studies have further suggested that meal timing and meal regularity may be associated with metabolic health, obesity-related outcomes, and healthy aging among older adults [17,18,19]. Behaviors such as breakfast skipping, infrequent meal consumption, and irregular eating schedules have been associated with obesity and metabolic disturbances [20,21,22,23,24] and often co-occur with other health-related behaviors, including physical inactivity, inadequate sleep, and smoking [20,25]. These considerations underscore the need to examine meal regularity as a potentially modifiable eating behavior associated with both general and abdominal obesity in older adults.

Obesity among older adults is not attributable to a single lifestyle factor; rather, it is associated with multiple demographic, socioeconomic, behavioral, and health-related factors [25,26]. Therefore, a comprehensive framework is needed to better understand the complex relationships among these factors and obesity in older adults.

Most previous studies have concentrated on populations such as children and adolescents [15,16,27,28], women [29], rural residents [30], or individuals with underweight [31,32], rather than specifically analyzing older adults. In addition, much of the existing research has primarily focused on associations between dietary habits and obesity, with less attention to organizing these factors within a theoretical framework. Although some studies have indicated that regular eating patterns are associated with decreased visceral fat accumulation [33], few studies have specifically examined meal regularity and its associations with both general and abdominal obesity among older adults. Moreover, limited attention has been given to meal regularity across breakfast, lunch, and dinner while considering the demographic, socioeconomic, behavioral, and health-related characteristics of older adults.

Andersen’s Behavioral Model provides an organizational framework for classifying relevant factors into predisposing, enabling, and need factors. In this study, the framework was used to organize factors associated with obesity and guide their interpretation.

Therefore, using nationally representative data from the 2019–2021 Korea National Health and Nutrition Examination Survey (KNHANES), this study aimed to examine the associations between meal regularity and general and abdominal obesity among Korean adults aged 65 years or older.

This study adds to the existing literature in several respects. First, it focuses on older adults, a population that has received comparatively less attention in previous studies examining meal regularity and obesity. Second, it examines meal regularity as a potentially modifiable eating behavior associated with general and abdominal obesity. Third, Andersen’s Behavioral Model was used as an organizational framework to classify obesity-related factors into predisposing, enabling, and need factors and to provide a structured context for interpreting these associations.

## 2. Methods

### 2.1. Study Design

This descriptive correlational study was conducted to examine the associations of predisposing, enabling, and need factors with general and abdominal obesity among Korean adults aged 65 years or older.

### 2.2. Data Source

The research employed raw data from the 8th Korea National Health and Nutrition Examination Survey (KNHANES VIII: 2019–2021) administered by the Korea Disease Control and Prevention Agency (KDCA). KNHANES is a triennial, nationally representative survey mandated under the National Health Promotion Act to assess the health and nutritional status of Korean citizens. The survey utilized a two-stage stratified cluster sampling design based on region, residential area, and housing type. Additional stratification accounted for household area ratio and the education level of the household head. Over the three years, 576 primary sampling units were selected, with 20 households chosen from each unit, excluding institutions and foreign households. All members aged one year or older within selected households participated. Data collection comprised physical examinations, health interviews, and nutrition surveys. To protect participants’ privacy, the Korea Disease Control and Prevention Agency anonymized all data. KNHANES employs a complex, multistage probability sampling design involving stratification, clustering, and weighting to ensure national representativeness. The survey sample consisted of 576 primary sampling units (PSUs) selected over 2019–2021 through a two-stage stratified cluster sampling process based on region, housing type, and household characteristics. Sampling weights provided by the Korea Disease Control and Prevention Agency were applied in all analyses to account for unequal selection probabilities, non-response, and post-stratification. Stratification variables and cluster variables were incorporated into the complex sample analysis procedures in IBM SPSS Statistics for Windows, version 26.0 (IBM Corp., Armonk, NY, USA).

### 2.3. Study Participants

**Figure 1:**
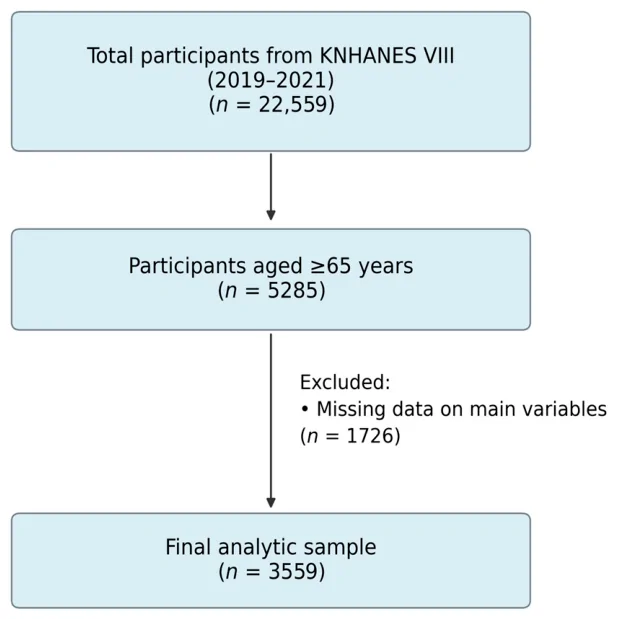
Flowchart of participant selection from the eighth Korea National Health and Nutrition Examination Survey (KNHANES VIII, 2019–2021).

Data from the 8th Korea National Health and Nutrition Examination Survey (KNHANES VIII, 2019–2021) were used for this study. Among 22,559 respondents, 5285 individuals aged 65 years or older were identified. After excluding participants with missing values for the main study variables, 3559 individuals were included in the final analysis. The weighted sample represented 5,476,279 older adults in the Korean population. (Figure 1).

Participants with missing data for any of the exposure, outcome, or covariate variables required for the analyses were excluded using a complete-case approach.

### 2.4. Theoretical Framework

Andersen’s Behavioral Model of Health Services Use was used as an organizational framework to classify the study variables and guide covariate selection and interpretation [34,35]. The model was not used to test causal pathways, mediation effects, or directional relationships between the study variables.

Based on this organizational framework, predisposing characteristics included age, sex, education level, and marital status. Household income was classified as an enabling factor, while hypertension and diabetes were classified as need factors. Smoking, alcohol consumption, physical activity, and the meal-consumption variables were considered health-behavior variables. The meal-consumption variables included regular breakfast, lunch, and dinner consumption and a composite indicator based on the number of meal types regularly consumed. General obesity and abdominal obesity were treated as health outcomes.

**Figure 2:**
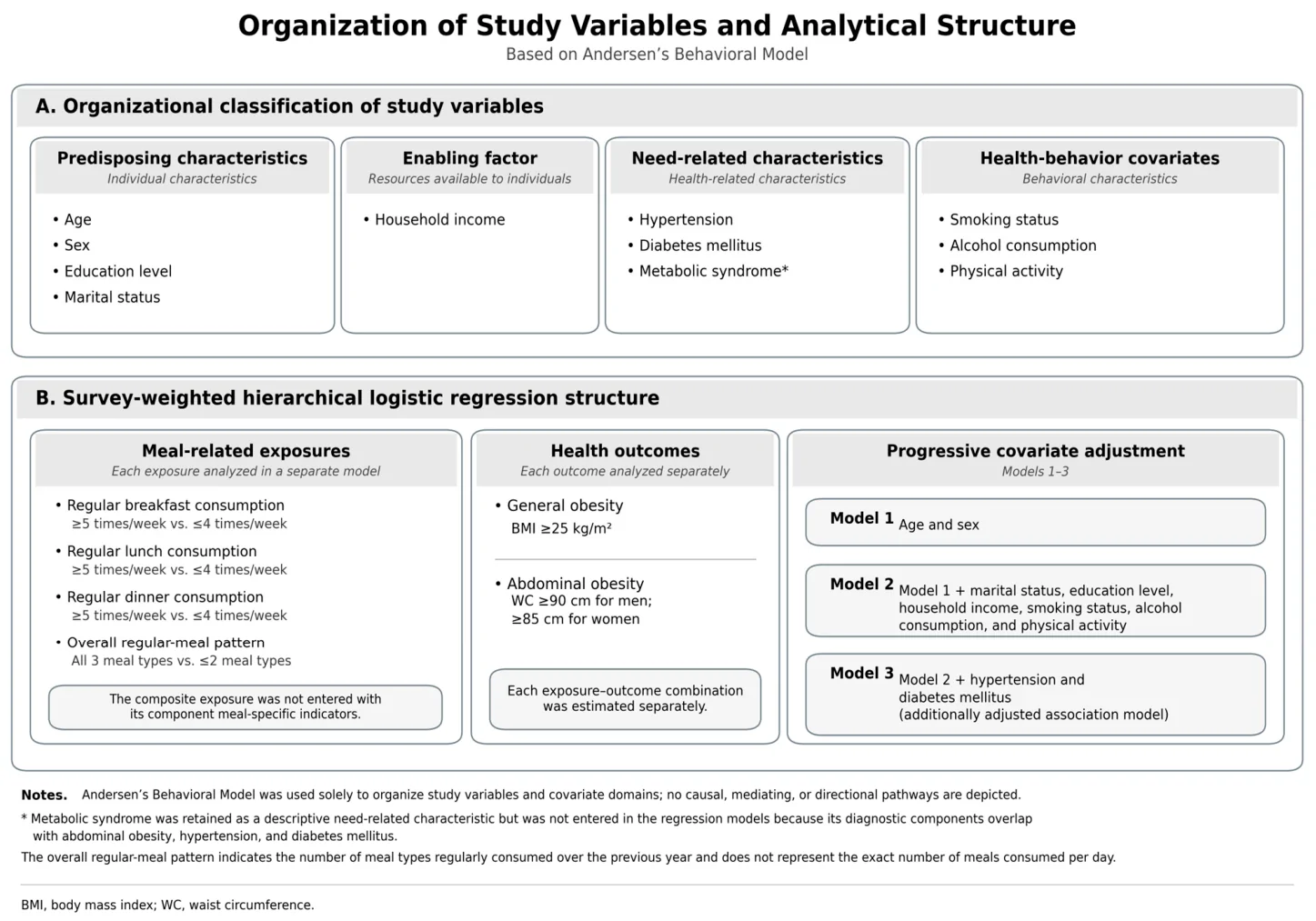
Organization of study variables and analytical structure based on Andersen’s Behavioral Model. No causal or mediating pathways are specified, and each meal-related exposure was examined in a separate survey-weighted logistic regression model. BMI, body mass index; WC, waist circumference.

The framework was used to organize the variables into conceptually relevant domains and to support the interpretation of the observed multivariable associations. Accordingly, the classification of variables within Andersen’s model should not be interpreted as evidence that predisposing, enabling, or need factors causally influence dietary behaviors or obesity outcomes. Rather, the framework provided a theory-informed structure for examining associations among sociodemographic characteristics, health-related characteristics, dietary behaviors, and obesity outcomes in Korean older adults [34,35] (Figure 2). 

### 2.5. Study Variables

#### 2.5.1. Dependent Variables

General obesity was evaluated through anthropometric assessments. Height, weight, and body mass index (BMI, body weight [kg]/height [m^2^]) were measured while participants wore light clothing. Height was recorded to the nearest 0.1 cm using a stadiometer (SECA 225, SECA, Hamburg, Germany), and weight was obtained to the nearest 0.1 kg utilizing a digital scale (GL-6000-20, CAS, Yangju, Republic of Korea). BMI was calculated according to the standard formula; obesity was defined as BMI ≥ 25 kg/m^2^ according to the WHO Asia-Pacific criteria for Asian populations and the Korean Society for the Study of Obesity guidelines. Asian populations tend to have higher cardiometabolic risk at lower BMI levels compared with Western populations, supporting the use of lower BMI thresholds in Korean adults.

Abdominal obesity was assessed using waist circumference (WC), measured to the nearest 0.1 cm with a measuring tape (SECA 200, SECA, Hamburg, Germany). WC was measured at the midpoint between the lower margin of the last palpable rib and the top of the iliac crest along the mid-axillary line after normal expiration. In accordance with the Korean Society for the Study of Obesity criteria for Korean adults, abdominal obesity was defined as a WC ≥ 90 cm for men and ≥85 cm for women. These criteria were consistently applied throughout all analyses of abdominal obesity.

#### 2.5.2. Independent Variables

Dietary behaviors were assessed using three self-reported meal-frequency items from the KNHANES VIII nutrition questionnaire. Participants were separately asked, “During the past year, how many times per week did you eat breakfast?”, “During the past year, how many times per week did you eat lunch?”, and “During the past year, how many times per week did you eat dinner?” The response options for each meal were 5–7 times per week, 3–4 times per week, 1–2 times per week, and almost never (0 times per week). Responses recorded as unknown or non-response were treated as missing.

For each meal, regular consumption was operationally defined as consumption 5–7 times per week and was coded as 1, whereas consumption four or fewer times per week was coded as 0. The ≥5-times-per-week cut-off corresponds to the highest predefined response category in the KNHANES questionnaire and indicates consumption on most days of the week. This approach uses the highest predefined KNHANES response category without assigning artificial numerical values to the ordinal response categories and is consistent with previous KNHANES-based studies [36,37].

A score representing the number of meal types regularly consumed was calculated by summing the three binary indicators for breakfast, lunch, and dinner, yielding a score ranging from 0 to 3. Accordingly, the score represents the number of meal types regularly consumed over the previous year and not the actual number of meals consumed on a particular day. For descriptive analyses, scores of 0 and 1 were combined into the ≤1-meal-type category because both indicated markedly limited regular consumption of the three meal types and their separate cell sizes were small, which could result in unstable survey-weighted estimates. Participants with a score of 2 were classified as regularly consuming two meal types, and those with a score of 3 as regularly consuming all three meal types. For the logistic regression analyses, the score was dichotomized into regular consumption of all three meal types versus two or fewer meal types, with the latter used as the reference category. This dichotomization was used to compare participants who regularly consumed all three meal types with those who did not regularly consume all three meal types.

These indicators were intended to capture meal regularity rather than overall dietary quality. They did not assess food selection, portion size, total energy intake, or macronutrient composition. Although KNHANES provides nutrient-intake estimates based on a 24 h dietary recall, these measures were not included because the present study focused specifically on habitual meal regularity and because the recall period differed from that of the meal-frequency questions. Therefore, regular meal consumption in this study should not be interpreted as indicating a nutritionally balanced or healthy diet.

#### 2.5.3. Control Variables

##### Sociodemographic Factors

Sociodemographic variables included age, sex, education level, household income, and marital status. Education categories were defined as ≤elementary school, ≤middle school, ≤high school, or ≥college. Household income was represented as equivalized household income (monthly household income/√household size) and categorized into quartiles (Q1–Q4). Marital status was distinguished as married, divorced/separated, widowed, or never married. Smoking status was ascertained with self-reported current smoking (current smoker vs. non-smoker). Alcohol intake was divided into three categories: “high-risk drinking” (≥2 times/week and ≥7 drinks for men or ≥5 drinks for women per occasion), “moderate drinking” (≥1 time/month but not high-risk), and “non-drinking” (<1 time/month). Physical activity was classified as ‘active’ if participants engaged in moderate-intensity physical activity for at least 30 min per session on three or more occasions per week, otherwise classified as “inactive.”

##### Disease Risk Factors and Prevalence

Health status and disease prevalence were assessed by combining anthropometric measurements with biochemical analyses of blood samples. In addition to height, weight, BMI, and WC, blood pressure (BP) was recorded to evaluate the presence of hypertension. BP was measured three times using an aneroid sphygmomanometer and stethoscope while the participant’s arm was supported at the level of the heart (average arm height: 83 cm for men, 81 cm for women). The mean value of the second and third BP readings was calculated for analysis. Hypertension was defined by a systolic BP ≥ 140 mmHg, diastolic BP ≥ 90 mmHg, or current use of antihypertensive medications.

For laboratory assessment, participants were instructed to fast from 7 p.m. the day prior to the survey (water allowed). Blood samples were obtained and measured using the ADVIA 650 (Siemens Healthcare Diagnostics, Tarrytown, NY, USA). Fasting blood sugar (FBS), glycated hemoglobin (HbA1c), total cholesterol (TC), triglycerides (TG), and high-density lipoprotein cholesterol (HDL-C) levels were determined. Diabetes was classified as FBS ≥ 126 mg/dL, HbA1c ≥ 6.5%, diagnosis by a physician, or current use of insulin or oral hypoglycemic medications.

The prevalence of metabolic syndrome was established based on meeting three or more of the following five criteria: FBS ≥ 110 mg/dL, WC > 90 cm for men or >85 cm for women, TG ≥ 150 mg/dL, HDL-C < 40 mg/dL for men or <50 mg/dL for women, and elevated BP (systolic ≥ 130 mmHg or diastolic ≥ 85 mmHg). Participants who met two or fewer criteria were categorized as not having metabolic syndrome.

### 2.6. Statistical Analysis

Because the current study utilized primary datasets from KNHANES, complex sample statistical techniques were employed to accommodate the survey’s multi-stage sampling structure.

All analyses incorporated sampling weights, stratification variables, and clustering variables provided by the Korea Disease Control and Prevention Agency and were conducted using the Complex Samples module in SPSS to account for the complex survey design. For the pooled analysis of KNHANES 2019–2021, combined survey weights for the three-year period were applied. Variance estimates were calculated using the Taylor series linearization method within the Complex Samples module of SPSS to appropriately account for the multistage stratified cluster sampling design of KNHANES.

The statistical analyses included the following actions:

- First, to determine whether the general characteristics of participants significantly varied with dietary habits and to identify associations between dietary patterns and both general and abdominal obesity, the Rao–Scott χ^2^ test was used alongside the general linear model (GLM) approach.
- Second, to investigate the associations between dietary behaviors and general and abdominal obesity, survey-weighted hierarchical logistic regression analyses were conducted. Regular breakfast consumption, regular lunch consumption, regular dinner consumption, and regular consumption of all three meal types were examined in separate regression models for each obesity outcome and each level of covariate adjustment. The indicator of regular consumption of all three meal types was not entered simultaneously with the individual meal-specific indicators. The estimates from the separately fitted models were presented together to facilitate comparison.
- Third, given the complex survey design, conventional goodness-of-fit statistics developed for ordinary logistic regression, including the standard Hosmer–Lemeshow test, were not applied. Model estimation was assessed by examining the estimability and standard errors of the regression coefficients. All non-reference parameters had finite coefficient and standard-error estimates, and no non-estimable coefficients were identified. Multicollinearity was evaluated using variance inflation factors, which ranged from 1.04 to 2.79.

The survey-weighted descriptive and regression analyses were performed using IBM SPSS Statistics for Windows, version 26.0 (IBM Corp., Armonk, NY, USA). Nominal statistical significance was defined as a two-tailed *p*-value < 0.05. The *p*-values and 95% confidence intervals reported in the regression results were not adjusted for multiplicity. As a post hoc assessment, the Benjamini–Hochberg false discovery rate (FDR) procedure was applied to the eight exposure–outcome comparisons in Model 3, comprising four dietary exposures evaluated against two obesity outcomes. These comparisons were treated as one testing family because Model 3 was the principal reporting model. The assessment used the rounded *p*-values reported in the regression results; values reported as *p* < 0.001 were entered conservatively as 0.001. The resulting adjusted *p*-values are therefore approximate and were evaluated against a threshold of 0.05. This assessment was restricted to the Model 3 exposure–outcome comparisons. As a sensitivity analysis, breakfast, lunch, and dinner frequency were each entered as a four-category predictor (5–7 times/week, 3–4 times/week, 1–2 times/week, and almost never) in separate survey-weighted logistic regression models with the Model 3 covariates, using almost never as the reference category. The composite indicator was not reclassified. These exploratory category-specific comparisons were reported with nominal *p*-values and 95% confidence intervals without multiplicity adjustment (Supplementary Table S4).

## 3. Results

### 3.1. Dietary Habits According to General Characteristics of Older Adults

To assess the general characteristics of the study subjects and to explore differences in these traits by dietary habits, categorical variables were analyzed using the Rao–Scott χ^2^ test, while continuous variables were assessed by the general linear model (GLM). The analysis yielded the following outcomes:

Age differed significantly according to the number of meal types regularly consumed (*p* < 0.001), with the mean age increasing across the categories. Sex was also significantly associated with the number of regularly consumed meal types (*p* < 0.001). Marital status differed significantly across the regular-meal categories (*p* < 0.001), with regular consumption of all three meal types being more common among married participants.

Smoking status was significantly associated with the number of meal types regularly consumed (*p* < 0.001), and the prevalence of smoking was lower among participants who regularly consumed all three meal types. BMI also differed significantly across the regular-meal categories (*p* = 0.003), and the prevalence of general obesity was lower among participants who regularly consumed all three meal types (*p* < 0.001). Although mean waist circumference did not differ significantly across the regular-meal categories, the prevalence of abdominal obesity differed significantly according to the number of meal types regularly consumed (*p* < 0.001) (Table 1).

Comparisons between participants included in and excluded from the main analysis are presented in Supplementary Table S3. Significant differences were observed in sex (*p* = 0.041), marital status (*p* < 0.001), household income (*p* = 0.010), alcohol consumption (*p* = 0.013), and metabolic syndrome (*p* < 0.001). No statistically significant differences were observed in the prevalence of general obesity (*p* = 0.207) or abdominal obesity (*p* = 0.541). These comparisons were based on the available observations for each characteristic (Supplementary Table S3).

### 3.2. Comparison of Dietary Habits by Obesity Status Among Older Adults

To analyze dietary habits according to obesity status among older adults, the Rao–Scott χ^2^ test was conducted. The findings were as follows:

When general obesity was classified based on BMI, significant differences were observed in regular breakfast consumption (*p* = 0.008), regular dinner consumption (*p* = 0.002), and the distribution of the number of regularly consumed meal types (*p* < 0.001). Participants with general obesity were less likely to consume breakfast and dinner at least five times per week and were less likely to regularly consume all three meal types than those without general obesity.

When abdominal obesity was classified based on waist circumference, significant differences were observed in regular lunch consumption (*p* = 0.012) and the distribution of the number of regularly consumed meal types (*p* = 0.003). Participants with abdominal obesity were less likely to consume lunch at least five times per week and were less likely to regularly consume all three meal types than those without abdominal obesity (Table 2).

**Figure 3:**
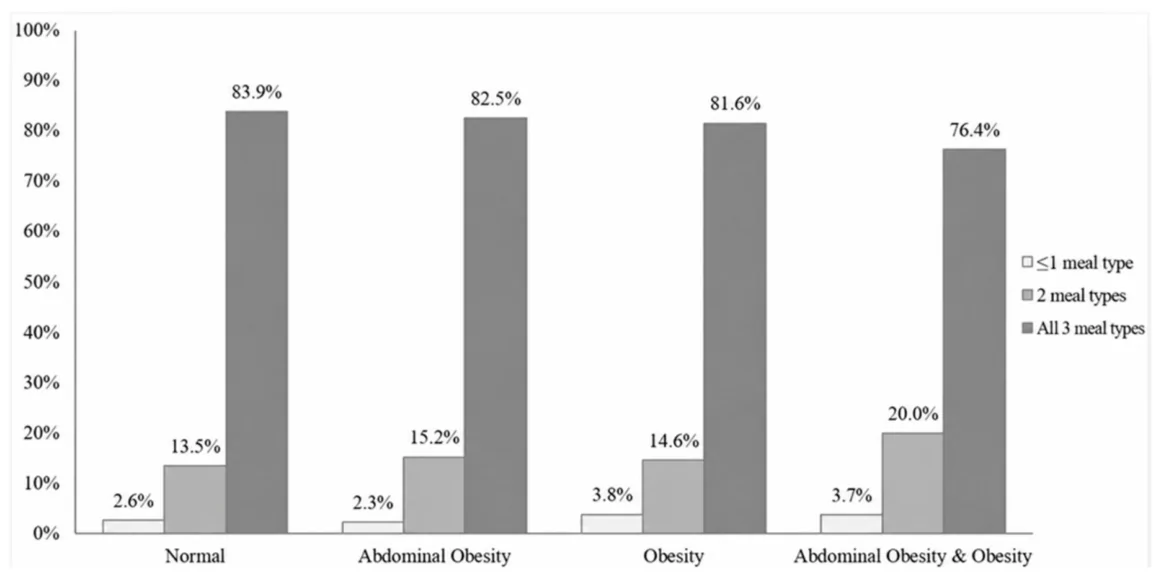
Distribution of the number of meal types regularly consumed according to general and abdominal obesity status.

Overall, the proportion of participants regularly consuming all three meal types was lower among those with general or abdominal obesity. Conversely, participants with obesity had higher proportions of regular consumption of two or fewer meal types. These categories represent the number of breakfast, lunch, and dinner meal types regularly consumed over the previous year rather than the actual number of meals consumed per day (Figure 3).

### 3.3. Associations Between Dietary Habits and Obesity in Older Adults

To examine the associations between dietary behaviors and obesity outcomes within Andersen’s Behavioral Model, survey-weighted hierarchical logistic regression analyses were conducted using a sequential domain-based adjustment strategy. Model 1 included age and sex as predisposing factors. Model 2 additionally included marital status and education as predisposing factors, household income as an enabling factor, and smoking, alcohol consumption, and physical activity as health-behavior covariates. Model 3 further included hypertension and diabetes mellitus as need factors. Regular breakfast, lunch, and dinner consumption and regular consumption of all three meal types were examined in separate regression models for each obesity outcome and each level of adjustment. The indicator of regular consumption of all three meal types was not entered simultaneously with the individual meal-specific indicators. The regression results are presented in Table 3.

Hypertension and diabetes mellitus were included as major chronic health conditions that may be associated with dietary behaviors and body weight because their diagnosis and management can be accompanied by changes in health and dietary behaviors. However, because these conditions may also occur downstream of obesity, Model 3 was interpreted as an additionally adjusted association model rather than as an estimate of the total causal effect. Metabolic syndrome was included in the theoretical framework and Table 1 as a descriptive need-related characteristic but was excluded from the regression models because its diagnostic components overlap with abdominal obesity, hypertension, and diabetes mellitus. Including metabolic syndrome could therefore have resulted in circular adjustment, overadjustment, and multicollinearity. The variance inflation factor values ranged from 1.04 to 2.79, indicating that multicollinearity was not a concern in the regression models.

The sequential models showed whether the associations between meal regularity and obesity outcomes persisted after accounting for predisposing, enabling, behavioral, and need-related characteristics.

For general obesity, regular breakfast consumption, regular dinner consumption, and regular consumption of all three meal types were significantly associated with lower odds of obesity in Models 1 and 2. In Model 3, after additional adjustment for hypertension and diabetes mellitus, regular breakfast consumption (odds ratio [OR] = 0.64, *p* = 0.008), regular lunch consumption (OR = 0.74, *p* = 0.042), regular dinner consumption (OR = 0.51, *p* = 0.001), and regular consumption of all three meal types (OR = 0.61, *p* < 0.001) were significantly associated with lower odds of general obesity. Among the separately estimated meal-specific associations, regular dinner consumption had the smallest odds ratio, corresponding to 49% lower odds of general obesity. Regular consumption of all three meal types was associated with 39% lower odds of general obesity compared with regular consumption of two or fewer meal types. The associations for regular breakfast consumption, regular dinner consumption, and regular consumption of all three meal types were nominally significant across Models 1–3. The association between regular lunch consumption and general obesity reached nominal significance only in Model 3 (*p* = 0.042) and did not remain significant in the post hoc FDR assessment (adjusted *p* ≈ 0.056). Full covariate estimates for the fully adjusted general-obesity models are presented in Supplementary Table S1.

**Figure 4:**
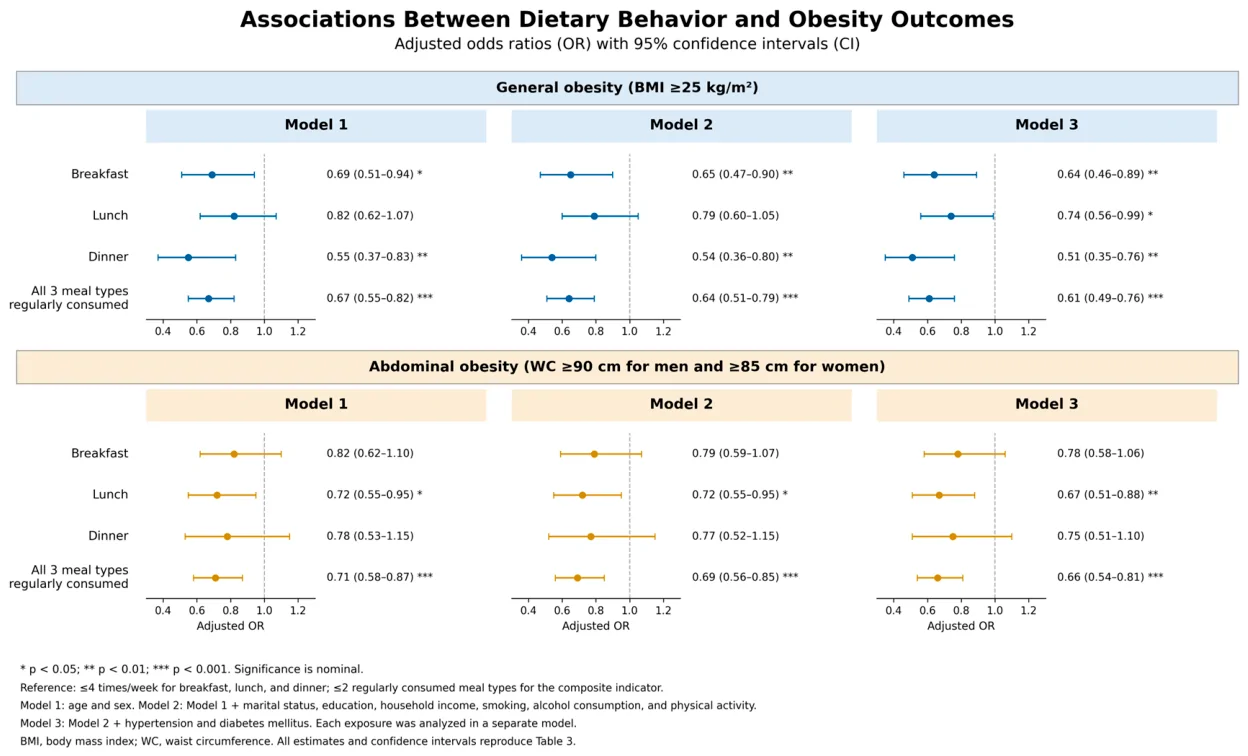
Forest plot of associations between dietary behaviors and obesity outcomes according to hierarchical logistic regression models. OR, odds ratio; CI, confidence interval; BMI, body mass index; WC, waist circumference.

For abdominal obesity, regular lunch consumption and regular consumption of all three meal types were significantly associated with lower odds across Models 1–3. In Model 3, which additionally adjusted for hypertension and diabetes mellitus, regular lunch consumption was associated with 33% lower odds of abdominal obesity (OR = 0.67, *p* = 0.004), and regular consumption of all three meal types was associated with 34% lower odds compared with regular consumption of two or fewer meal types (OR = 0.66, *p* < 0.001). Regular breakfast and dinner consumption were not significantly associated with abdominal obesity in any of the models (Figure 4). Full covariate estimates for the fully adjusted abdominal-obesity models are presented in Supplementary Table S2.

In the four-category sensitivity analysis, lunch consumption 5–7 times/week versus almost never was nominally associated with lower odds of general obesity (adjusted odds ratio [aOR] = 0.61, 95% confidence interval [CI]: 0.41–0.92; *p* = 0.017) and abdominal obesity (aOR = 0.59, 95% CI: 0.38–0.92; *p* = 0.019). Breakfast and dinner consumption 5–7 times/week versus almost never were not statistically significant for either outcome in Model 3 (Supplementary Table S4). These exploratory comparisons used a different reference category from the primary dichotomous analyses and were not adjusted for multiplicity.

## 4. Discussion

Using nationally representative KNHANES VIII data, this study found that regular meal consumption was associated with lower odds of general and abdominal obesity among Korean older adults after sequential adjustment for the domains specified in Andersen’s Behavioral Model. Regular breakfast and dinner consumption were associated with lower odds of general obesity, whereas regular lunch consumption was associated with lower odds of abdominal obesity. The association between regular lunch consumption and general obesity was nominally significant only in Model 3 and did not remain significant after the post hoc FDR adjustment.

Regular consumption of all three meal types was associated with 39% lower odds of general obesity (OR = 0.61) and 34% lower odds of abdominal obesity (OR = 0.66). These findings suggest that meal regularity may represent a modifiable health behavior related to obesity outcomes in later life, although the cross-sectional design precludes causal interpretation.

Household income was classified as an enabling factor because financial resources may influence the ability to purchase food, prepare meals, access transportation, and use community meal services. Although household income was not significantly associated with meal regularity in the descriptive analysis, its inclusion in the analytical framework allowed the associations between meal consumption and obesity to be interpreted after accounting for measured socioeconomic resources. From this perspective, community dining, home-delivered meals, food-assistance programs, and transportation support may be considered potential enabling strategies for supporting regular meal consumption. However, these strategies were not directly evaluated in the present study. Future longitudinal and intervention studies are needed to determine whether such strategies improve meal regularity or obesity-related outcomes among older adults.

Smoking, alcohol consumption, physical activity, and meal consumption were treated as health behaviors within the analytical structure. The lower prevalence of smoking among participants who regularly consumed all three meal types suggests that meal regularity may cluster with other health-promoting behaviors. Previous studies have similarly linked meal skipping with smoking and less favorable lifestyle patterns [38,39,40]. This clustering may occur because nicotine suppresses appetite or because smoking and irregular eating share broader behavioral and social determinants. Therefore, the observed association between meal regularity and obesity may partly reflect overall lifestyle stability rather than meal regularity alone.

The supplementary analyses also showed lower adjusted odds of general obesity among current smokers. This finding requires cautious interpretation. Residual confounding by dietary intake, underlying illness, and frailty, together with differences in associated health behaviors, may have contributed to the observed association. In addition, the current-smoking classification did not distinguish former smokers from never smokers within the non-current-smoking group, so weight changes following smoking cessation may have influenced the comparison. Selective survival among older adults may also have affected the observed association. These potential explanations could not be established in the present cross-sectional analysis. Accordingly, the lower odds of general obesity among current smokers should not be interpreted as evidence of a health benefit or as support for smoking as a weight-control strategy.

Hypertension and diabetes mellitus were included as need factors representing chronic health conditions that may influence both dietary behavior and body weight. The associations between regular meal consumption and obesity remained after these need factors were added to the fully adjusted model. This suggests that meal regularity was associated with obesity outcomes beyond the measured disease-related characteristics. However, hypertension and diabetes may also be consequences of obesity or may lead older adults to modify their diets. Accordingly, these variables should be interpreted as additionally adjusted need factors rather than as evidence of a causal pathway.

Several physiological and behavioral mechanisms may explain the association between regular meals and general obesity. Skipping meals may prolong fasting and promote compensatory overeating, increased snacking, or the selection of energy-dense foods later in the day [41,42]. A predictable meal schedule may support appetite regulation and a more even distribution of energy intake, which may be particularly relevant for older adults with reduced basal metabolic rates and lower physical activity [8,9,10]. Nevertheless, the present study did not assess portion size, total energy intake, meal timing, or dietary quality. Therefore, these mechanisms should be regarded as plausible explanations rather than directly tested pathways.

The meal-specific findings also warrant cautious interpretation. Regular lunch consumption was associated with lower odds of abdominal obesity, whereas the lowest odds ratio for general obesity was observed for regular dinner consumption. A regular midday meal may reduce prolonged daytime fasting, afternoon snacking, and compensatory intake later in the evening, thereby supporting glucose regulation and limiting behaviors associated with visceral fat accumulation [19,21,32,33]. Regular dinner consumption may indicate a stable daily routine and may reduce unstructured late-night eating or reliance on snacks after an omitted evening meal. It may also reflect social support, functional independence, or the ability to prepare meals consistently. However, breakfast, lunch, and dinner were analyzed in separate regression models; therefore, differences in the magnitude of the odds ratios do not establish that one meal is more protective than another.

The different findings for general and abdominal obesity may also reflect differences between the two outcomes. BMI represents overall body mass relative to height, whereas waist circumference more directly reflects central fat distribution [42,43]. Meal-related behaviors may therefore show different associations with these obesity phenotypes [36]. However, neither BMI nor waist circumference directly measures visceral adiposity or age-related loss of muscle mass, and the findings should not be interpreted as evidence that regular lunch or dinner consumption selectively alters a specific fat compartment.

Taken together, Andersen’s Behavioral Model provides a structured interpretation of the findings. Predisposing factors may shape meal routines, enabling resources may support access to regular meals, need factors may influence dietary modification, and meal regularity may represent a potentially modifiable health behavior associated with obesity outcomes [29,34]. This interpretation suggests potential avenues for future research at multiple levels. Future studies could evaluate whether screening for meal skipping and related barriers is useful in clinical and community settings and whether food-access programs, congregate dining, meal-delivery services, and transportation support can improve meal regularity and related health outcomes among nutritionally vulnerable older adults [43,44].

These associations should nevertheless be interpreted cautiously. The cross-sectional design does not establish temporality, and reverse causation is possible because older adults with obesity or chronic diseases may intentionally modify their meal patterns. In addition, total energy intake, living arrangement, oral health, functional status, and other relevant factors were not included in the adjusted models. Future longitudinal and intervention studies should examine how predisposing, enabling, and need factors interact with meal regularity over time and whether improving meal regularity contributes to changes in general or abdominal obesity.

## 5. Conclusions

This study performed a secondary analysis using raw data from the 8th Korea National Health and Nutrition Examination Survey (2019–2021), comprising 3559 adults aged 65 years and above. Andersen’s Behavioral Model of Health Services Use served as the conceptual foundation for investigating the associations of dietary behaviors on obesity and abdominal obesity in the elderly population. The results demonstrated that demographic characteristics, including age, gender, and marital status, were significantly related to dietary practices. In addition, older adults who adhered to regular meal routines experienced lower rates of smoking, reduced body mass index (BMI), and decreased prevalence of abdominal obesity.

In the fully adjusted models, regular breakfast consumption, regular dinner consumption, and regular consumption of all three meal types were associated with lower odds of general obesity. The association between regular lunch consumption and general obesity was nominally significant but did not remain significant after the post hoc FDR adjustment.

For abdominal obesity, only regular lunch consumption and regular consumption of all three meal types were significantly associated with lower odds. Regular consumption of all three meal types was associated with 39% lower odds of general obesity and 34% lower odds of abdominal obesity compared with regular consumption of two or fewer meal types. These findings should be interpreted as associations rather than causal effects.

Meal regularity may be considered a behavioral factor warranting further investigation in relation to obesity among older adults. Community-centered approaches, including congregate meal programs, meal-delivery services, and social dining opportunities, were not evaluated in this study. Their potential effects on meal regularity and obesity outcomes require evaluation in longitudinal and intervention studies.

## 6. Limitations

This study has several limitations. First, because of its cross-sectional design, causal relationships between meal regularity and obesity could not be established, and reverse causation remains possible. Older adults with obesity or chronic diseases may have intentionally modified their meal patterns for weight management or treatment.

Second, approximately one third of the eligible older adults were excluded because of incomplete data. Of the 5285 eligible participants, 1726 participants (32.7%) were excluded, and a complete-case approach was used for the analysis. Consequently, the final analytic sample may have differed systematically from the excluded participants, potentially introducing selection bias. Although the official KNHANES complex-sample weights were applied, these weights may not fully account for selection associated with item-level missingness. Therefore, the findings should be interpreted cautiously.

Third, the assessment of dietary behavior was limited to self-reported questions regarding the frequency and regularity of breakfast, lunch, and dinner over the previous year. These measures were designed to assess one specific behavioral dimension—habitual meal regularity—and should not be regarded as comprehensive indicators of dietary quality or healthfulness. The broad categorical responses may also have been affected by recall and social desirability bias and did not capture food selection, portion size, exact meal timing, total energy intake, overall dietary quality, or macronutrient composition. Consequently, participants classified as regularly consuming meals may not necessarily have consumed nutritionally balanced or healthy diets, and the observed associations may partly reflect unaccounted-for differences in dietary quality or nutrient intake rather than meal regularity alone. Although KNHANES provides nutrient-intake estimates derived from a 24 h dietary recall, these estimates were not included because their reference period differed from that of the past-year meal-frequency questions and a single-day recall may not adequately represent usual individual intake. Furthermore, because of the cross-sectional design, the roles of total energy intake and dietary quality as potential confounding or intermediate factors could not be clearly distinguished. Future studies should combine meal-regularity indicators with repeated dietary recalls, validated dietary-quality indices, total energy intake, and macronutrient composition.

Fourth, residual confounding may remain because several relevant factors, including total energy intake, overall dietary quality, living arrangement, oral health, functional status, frailty, depressive symptoms, medication use, and other chronic conditions, were not included in the adjusted models. These unaccounted-for factors may be associated with both meal regularity and obesity status and may therefore partly explain the observed associations. Accordingly, the reported associations should not be interpreted as independent of these factors. Hypertension and diabetes mellitus may also function as obesity-related consequences, raising the possibility of overadjustment in Model 3.

Fifth, BMI and waist circumference do not directly measure visceral adiposity, muscle mass, or sarcopenic obesity. These limitations are particularly relevant in older adults because age-related loss of muscle mass and changes in fat distribution may result in misclassification of obesity status. In addition, the findings may not be fully generalizable to populations with different cultural and dietary contexts. Future longitudinal studies should incorporate more detailed nutritional and functional assessments as well as direct measures of body composition and visceral adiposity.

Sixth, the statistical evaluation of the regression models was incomplete. Although Nagelkerke pseudo-R^2^ values and F statistics are presented in Table 3, additional survey-design-appropriate assessments of calibration and discrimination were not performed. Finite coefficient and standard-error estimates and the absence of substantial multicollinearity do not establish adequate overall model fit. Accordingly, the adjusted associations should be interpreted cautiously, as their robustness has not been established through comprehensive model diagnostics.

Seventh, subgroup analyses stratified by sex or age were not conducted. Although sex and age were included as covariates in all regression models, statistical adjustment does not exclude the possibility that the associations between meal regularity and obesity may differ according to sex or age group. Moreover, only 96 participants regularly consumed one or fewer meal types, and stratification could have resulted in small effective sample sizes and imprecise estimates under the KNHANES complex survey design. Therefore, the present findings should be interpreted as population-average associations among Korean adults aged 65 years or older. Future studies with sufficient sample sizes within meal-pattern categories should formally evaluate effect modification by sex and age using interaction terms and survey-design-appropriate variance estimation.

Eighth, there is a possibility of selection bias due to significant differences in gender, marital status, household income, alcohol consumption and metabolic syndrome between the excluded non-participants—who were omitted owing to missing data in key covariates—and the final analytical sample (*p* < 0.05). Although we applied complex sampling weights to maintain national representativeness, future studies should prioritize strategies to minimize missing covariate data during the study design and data collection stages.

Ninth, regular lunch consumption was associated with lower odds of general obesity at the nominal significance level in Model 3 (*p* = 0.042), but this association did not remain statistically significant in the post hoc FDR assessment based on the reported *p*-values (adjusted *p* ≈ 0.056). This suggests that the relationship between lunch consumption and general obesity may be a borderline finding susceptible to multiplicity adjustment, warranting a cautious interpretation of this result. Future studies are needed to further verify the consistency of this association.

## 7. Implications

First, this research provided a comprehensive assessment of associations between general characteristics, health behaviors, and dietary habits in older adults, identifying determinants linked to both obesity and abdominal obesity.

Second, the application of Andersen’s Behavioral Model indicates that habitual meal regularity should be interpreted within broader sociodemographic, behavioral, and health-related contexts. However, the present findings do not establish that modifying meal regularity would lead to reductions in obesity.

Third, regular consumption of all three meal types was associated with lower BMI and lower odds of abdominal obesity among older adults. This finding identifies meal regularity as a potential behavioral factor for further investigation rather than as an intervention whose effectiveness has been demonstrated.

Fourth, congregate meal programs, home meal-delivery services, food-access programs, social dining opportunities, and transportation support were not directly evaluated in the present study. These approaches should therefore be regarded as potential strategies warranting further evaluation in longitudinal and intervention studies, particularly among older adults who live alone or experience difficulties accessing or preparing meals.

## Figures and Tables

**Table 1:** General characteristics of the participants (N = 3559).

Key Characteristics	Classification Categories	Number of Meal Types Regularly Consumed			*p*
		≤1 (*n* = 96)	2 (*n* = 541)	All 3 (*n* = 2922)	
Age Group		71.44 ± 0.53	71.75 ± 0.25	72.70 ± 0.14	<0.001 ***
Gender	Male	23.0 (4.9)	36.7 (2.1)	46.6 (0.9)	<0.001 ***
	Female	77.0 (4.9)	63.3 (2.1)	53.4 (0.9)	
Marital status	Married	42.5 (6.0)	54.1 (2.7)	71.2 (1.1)	<0.001 ***
	Divorced/Separated	14.6 (3.8)	11.2 (1.4)	5.0 (0.5)	
	Widowed	42.4 (6.0)	32.8 (2.4)	23.0 (1.0)	
	Unmarried	0.5 (0.5)	1.9 (0.7)	0.8 (0.2)	
Education	Elementary school	59.7 (5.8)	51.0 (2.5)	50.0 (1.4)	0.111
	Middle school	17.3 (4.9)	18.1 (1.8)	17.9 (0.8)	
	High school	19.6 (4.3)	22.3 (2.2)	19.9 (0.9)	
	≥College	3.4 (1.9)	8.6 (1.3)	12.2 (1.0)	
Household income	Low	44.4 (6.2)	44.6 (2.7)	40.8 (1.4)	0.624
	Mid-low	30.8 (5.6)	29.3 (2.6)	29.4 (1.1)	
	Mid-high	18.1 (5.3)	15.0 (1.9)	18.4 (1.1)	
	High	6.7 (3.0)	11.0 (1.6)	11.5 (0.9)	
Smoking	Yes	21.4 (5.4)	14.3 (1.9)	8.5 (0.6)	<0.001 ***
	No	78.6 (5.4)	85.7 (1.9)	91.5 (0.6)	
Alcohol consumption	High-risk alcohol use	4.7 (2.3)	7.7 (1.5)	5.0 (0.5)	0.191
	Moderate alcohol use	26.1 (5.2)	26.0 (2.1)	29.1 (1.0)	
	Abstinence from alcohol	69.1 (5.4)	66.3 (2.4)	65.9 (1.1)	
Physical activity	Yes	11.0 (3.9)	11.6 (1.6)	13.5 (0.8)	0.511
	No	89.0 (3.9)	88.4 (1.6)	86.5 (0.8)	
HTN	Yes	60.0 (6.1)	62.2 (2.3)	62.3 (1.1)	0.725
	No	40.0 (6.1)	37.8 (2.3)	37.7 (1.1)	
DM	Yes	21.7 (5.1)	28.4 (2.3)	30.3 (0.9)	0.821
	No	78.3 (5.1)	71.6 (2.3)	69.7 (0.9)	
Metabolic syndrome	Yes	21.4 (4.4)	33.6 (2.3)	31.3 (1.1)	0.103
	No	78.6 (4.4)	66.4 (2.3)	68.7 (1.1)	
Obesity	BMI (kg/m2)	24.61 ± 0.55	24.66 ± 0.16	24.08 ± 0.07	0.003 **
	Yes	45.8 (6.1)	44.9 (2.3)	34.8 (1.0)	<0.001 ***
	No	54.2 (6.1)	55.1 (2.3)	65.2 (1.0)	
Abdominal obesity	WC (cm)	87.87 ± 1.47	88.14 ± 0.47	87.17 ± 0.20	0.145
	Yes	55.3 (5.9)	58.7 (2.3)	49.6 (1.1)	<0.001 ***
	No	44.7 (5.9)	41.3 (2.3)	50.4 (1.1)	

** *p* < 0.01; *** *p* < 0.001. Values are presented as weighted percentages (standard errors) or means ± standard errors. N, total sample size; n, subgroup sample size; SE, standard error; BMI, body mass index; WC, waist circumference; HTN, hypertension; DM, diabetes mellitus.

**Table 2:** Dietary behavior according to obesity and abdominal obesity.

Variables	Obesity		*p*	Abdominal Obesity		*p*
	Yes	No		Yes	No	
Breakfast	89.4 (1.1)	92.7 (0.7)	0.008 **	90.7 (0.8)	92.2 (0.8)	0.199
Lunch	89.7 (1.1)	91.5 (0.8)	0.115	89.4 (1.0)	92.3 (0.7)	0.012 *
Dinner	93.7 (0.9)	96.5 (0.5)	0.002 **	94.9 (0.6)	96.1 (0.6)	0.160
Number of meal types regularly consumed						
≤1 meal type	3.7 (0.7)	2.5 (0.4)	<0.001 ***	3.2 (0.5)	2.7 (0.5)	0.003 **
2 meal types	19.5 (1.4)	14.0 (0.9)		18.3 (1.2)	13.5 (1.0)	
All 3 meal types	76.8 (1.5)	83.5 (1.0)		78.5 (1.3)	83.7 (1.1)	

* *p* < 0.05; ** *p* < 0.01; *** *p* < 0.001. Values are presented as weighted percentages (standard errors). SE, standard error. Meal types refer to breakfast, lunch, and dinner consumed regularly 5–7 times per week.

**Table 3:** Association between dietary behavior and obesity and abdominal obesity.

**Independent Variables**	**Dependent Variable: Obesity**							
	**Model 1**		**Model 2**		**Model 3**		**Nagelkerke R^2^**	**F**
	**OR (95% CI)**	***p***	**OR (95% CI)**	*p*	**OR (95% CI)**	***p***		
Breakfast	0.69 (0.51–0.94)	0.019 *	0.65 (0.47–0.90)	0.009 **	0.64 (0.46–0.89)	0.008 **	0.075	8.14 ***
Lunch	0.82 (0.62–1.07)	0.148	0.79 (0.60–1.05)	0.107	0.74 (0.56–0.99)	0.042 *	0.072	7.74 ***
Dinner	0.55 (0.37–0.83)	0.004 **	0.54 (0.36–0.80)	0.002 **	0.51 (0.35–0.76)	0.001 **	0.076	7.88 ***
All 3 meal types regularly consumed	0.67 (0.55–0.82)	<0.001 ***	0.64 (0.51–0.79)	<0.001 ***	0.61 (0.49–0.76)	<0.001 ***	0.081	8.46 ***
**Independent Variables**	**Dependent Variable: Abdominal Obesity**							
	**Model 1**		**Model 2**		**Model 3**		**Nagelkerke R^2^**	**F**
	**OR (95% CI)**	***p***	**OR (95% CI)**	***p***	**OR (95% CI)**	***p***		
Breakfast	0.82 (0.62–1.10)	0.183	0.79 (0.59–1.07)	0.125	0.78 (0.58–1.06)	0.109	0.082	9.74 ***
Lunch	0.72 (0.55–0.95)	0.018 *	0.72 (0.55–0.95)	0.018 *	0.67 (0.51–0.88)	0.004 **	0.085	9.64 ***
Dinner	0.78 (0.53–1.15)	0.215	0.77 (0.52–1.15)	0.199	0.75 (0.51–1.10)	0.140	0.082	9.41 ***
All 3 meal types regularly consumed	0.71 (0.58–0.87)	<0.001 ***	0.69 (0.56–0.85)	<0.001 ***	0.66 (0.54–0.81)	<0.001 ***	0.088	10.23 ***

* *p* < 0.05; ** *p* < 0.01; *** *p* < 0.001. OR, odds ratio; CI, confidence interval; HTN, hypertension; DM, diabetes mellitus. Boldface denotes headings only. Reference: ≤4 times/week for each meal; ≤2 regularly consumed meal types for the composite indicator. Model 1: sex and age. Model 2: additionally marital status, education, household income, smoking, alcohol consumption, and physical activity. Model 3: additionally HTN and DM. Each exposure was analyzed in a separate regression model; estimates are presented together.

## Data Availability

The datasets analyzed and/or generated during this study can be accessed in the Korea Disease Control and Prevention Agency repository [https://knhanes.kdca.go.kr]. Additionally, the datasets are obtainable from the corresponding author upon reasonable request.

## Supplementary Materials

The following supporting information can be downloaded at: https://www.mdpi.com/article/10.3390/healthcare14193278/s1, Table S1: Full covariate estimates for general obesity in fully adjusted models; Table S2: Full covariate estimates for abdominal obesity in fully adjusted models; Table S3: Comparison of characteristics between included and excluded participants aged ≥65 years in KNHANES VIII (2019–2021); Table S4: Sensitivity analysis using the original four meal-frequency categories and Model 3 adjustment among Korean older adults (N = 3559).

## Abbreviations

The following abbreviations are used in this manuscript:

KNHANES	Korea National Health and Nutrition Examination Survey
KDCA	Korea Disease Control and Prevention Agency
BMI	Body Mass Index
WC	Waist Circumference
BP	Blood Pressure
FBS	Fasting Blood Sugar
HDL-C	High-Density Lipoprotein Cholesterol
aOR	Adjusted odds ratio
CI	Confidence interval
DM	Diabetes mellitus
FDR	False discovery rate
GLM	General linear model
HbA1c	Glycated hemoglobin
HTN	Hypertension
IRB	Institutional Review Board
OR	Odds ratio
PSU	Primary sampling unit
SE	Standard error
TC	Total cholesterol
TG	Triglycerides
WHO	World Health Organization
