# Supplementary material for: Association Between Meal Regularity and Obesity Among Korean Older Adults: A Population-Based Study Using KNHANES VIII
Source: Healthcare (Basel). 2026 Oct 2;14(19):3278. doi: 10.3390/healthcare14193278 (PMC13665403; doi:10.3390/healthcare14193278)
Supplement: Supplementary file 1 [file healthcare-14-03278-s001.zip › healthcare-4561969-supplementary.pdf]

Table S1. Full covariate estimates for general obesity in fully adjusted models

Outcome: general obesity (BMI ≥25 kg/m²). Each dietary exposure was analyzed in a separate Model 3.

Covariate	Comparison	Breakfast model		Lunch model		Dinner model		Overall pattern model	
		aOR (95% CI)	*p*	aOR (95% CI)	*p*	aOR (95% CI)	*p*	aOR (95% CI)	*p*
**Dietary exposure**	Model-specific contrast†	0.64 (0.46-0.89)	0.008	0.74 (0.56-0.99)	0.042	0.51 (0.35-0.76)	0.001	0.61 (0.49-0.76)	<0.001
**Age**	Per 1-year increase	0.96 (0.95-0.98)	<0.001	0.96 (0.94-0.98)	<0.001	0.96 (0.94-0.98)	<0.001	0.97 (0.95-0.98)	<0.001
**Sex**	Male vs. female	1.01 (0.83-1.23)	0.890	1.01 (0.83-1.22)	0.954	1.02 (0.84-1.25)	0.808	1.03 (0.85-1.26)	0.738
**Marital status**	Married vs. never married	1.44 (0.71-2.94)	0.313	1.36 (0.66-2.82)	0.402	1.33 (0.64-2.76)	0.440	1.47 (0.72-2.97)	0.288
	Divorced/separated vs. never married	1.06 (0.50-2.25)	0.877	1.04 (0.48-2.24)	0.927	0.98 (0.45-2.13)	0.966	1.03 (0.49-2.19)	0.932
	Widowed vs. never married	1.44 (0.69-3.01)	0.327	1.39 (0.66-2.94)	0.389	1.35 (0.64-2.87)	0.430	1.43 (0.69-2.96)	0.339
**Education level**	Elementary school or less vs. college or higher	1.28 (0.97-1.69)	0.086	1.26 (0.95-1.67)	0.104	1.27 (0.96-1.68)	0.098	1.26 (0.95-1.67)	0.105
	Middle school vs. college or higher	1.11 (0.81-1.52)	0.523	1.10 (0.80-1.51)	0.542	1.11 (0.81-1.52)	0.535	1.10 (0.80-1.51)	0.559
	High school vs. college or higher	0.91 (0.68-1.22)	0.516	0.91 (0.67-1.22)	0.519	0.91 (0.67-1.22)	0.516	0.89 (0.66-1.20)	0.445
**Household income**	Low vs. high	0.91 (0.67-1.23)	0.522	0.88 (0.65-1.18)	0.384	0.87 (0.65-1.18)	0.375	0.88 (0.65-1.19)	0.404
	Middle-low vs. high	0.98 (0.73-1.31)	0.878	0.95 (0.71-1.27)	0.721	0.94 (0.70-1.27)	0.688	0.95 (0.71-1.28)	0.744
	Middle-high vs. high	0.91 (0.66-1.26)	0.587	0.90 (0.65-1.24)	0.499	0.88 (0.64-1.21)	0.422	0.90 (0.65-1.25)	0.539
**Current smoking**	Yes vs. no	0.68 (0.50-0.91)	0.011	0.68 (0.50-0.92)	0.012	0.69 (0.51-0.93)	0.016	0.65 (0.48-0.88)	0.006
**Alcohol consumption**	High-risk drinking vs. abstinence	0.95 (0.63-1.43)	0.814	0.96 (0.64-1.45)	0.857	0.96 (0.64-1.43)	0.834	0.93 (0.62-1.40)	0.727
	Moderate drinking vs. abstinence	1.16 (0.95-1.42)	0.140	1.17 (0.96-1.43)	0.119	1.16 (0.95-1.42)	0.154	1.16 (0.95-1.42)	0.138
**Physical activity**	Active vs. inactive	0.93 (0.72-1.21)	0.587	0.93 (0.72-1.21)	0.595	0.93 (0.71-1.21)	0.594	0.93 (0.72-1.21)	0.607
**Hypertension**	Yes vs. no	2.24 (1.86-2.68)	<0.001	2.25 (1.87-2.70)	<0.001	2.24 (1.86-2.69)	<0.001	2.26 (1.88-2.71)	<0.001
**Diabetes mellitus**	Yes vs. no	1.44 (1.23-1.68)	<0.001	1.44 (1.23-1.68)	<0.001	1.44 (1.24-1.69)	<0.001	1.45 (1.24-1.70)	<0.001

Values are adjusted odds ratios (aORs) with 95% confidence intervals from survey-weighted logistic regression.
Breakfast, lunch, dinner, and the overall regular-meal pattern were analyzed in separate regression models; estimates from the separate models are presented together for comparison.
All Model 3 analyses were adjusted for age, sex, marital status, education level, household income, current smoking, alcohol consumption, physical activity, hypertension, and diabetes mellitus.
Reference outcome category: no obesity. Reference covariate categories are shown in the Comparison column.
†For the breakfast, lunch, and dinner models, regular consumption was defined as 5-7 times/week and compared with ≤4 times/week. For the overall-pattern model, regular consumption of all three meal types was compared with regular consumption of two or fewer meal types.
Abbreviations: aOR, adjusted odds ratio; BMI, body mass index; CI, confidence interval; WC, waist circumference.

Table S2. Full covariate estimates for abdominal obesity in fully adjusted models

Outcome: abdominal obesity, defined as WC ≥90 cm for men and ≥85 cm for women. Each dietary exposure was analyzed in a separate Model 3.

Covariate	Comparison	Breakfast model		Lunch model		Dinner model		Overall pattern model	
		aOR (95% CI)	*p*	aOR (95% CI)	*p*	aOR (95% CI)	*p*	aOR (95% CI)	*p*
**Dietary exposure**	Model-specific contrast†	0.78 (0.58-1.06)	0.109	0.67 (0.51-0.88)	0.004	0.75 (0.51-1.10)	0.140	0.66 (0.54-0.81)	<0.001
**Age**	Per 1-year increase	1.00 (0.98-1.02)	0.975	1.00 (0.98-1.02)	0.991	1.00 (0.98-1.02)	0.964	1.00 (0.98-1.02)	0.820
**Sex**	Male vs. female	0.85 (0.70-1.04)	0.106	0.85 (0.70-1.04)	0.110	0.85 (0.70-1.04)	0.111	0.87 (0.71-1.06)	0.160
**Marital status**	Married vs. never married	0.87 (0.42-1.81)	0.715	0.86 (0.42-1.77)	0.687	0.84 (0.40-1.76)	0.644	0.90 (0.45-1.83)	0.780
	Divorced/separated vs. never married	0.62 (0.28-1.37)	0.239	0.62 (0.29-1.34)	0.222	0.61 (0.27-1.34)	0.217	0.62 (0.29-1.32)	0.210
	Widowed vs. never married	0.81 (0.38-1.72)	0.579	0.80 (0.38-1.67)	0.547	0.79 (0.37-1.68)	0.534	0.81 (0.39-1.68)	0.572
**Education level**	Elementary school or less vs. college or higher	1.35 (1.01-1.81)	0.045	1.33 (0.99-1.78)	0.055	1.35 (1.00-1.81)	0.046	1.34 (1.00-1.80)	0.053
	Middle school vs. college or higher	1.18 (0.87-1.61)	0.290	1.17 (0.86-1.59)	0.308	1.18 (0.87-1.60)	0.290	1.17 (0.86-1.59)	0.315
	High school vs. college or higher	0.89 (0.67-1.19)	0.433	0.88 (0.66-1.18)	0.404	0.89 (0.67-1.19)	0.439	0.87 (0.65-1.17)	0.363
**Household income**	Low vs. high	1.03 (0.76-1.40)	0.834	1.00 (0.74-1.36)	0.999	1.01 (0.75-1.37)	0.929	1.01 (0.75-1.37)	0.939
	Middle-low vs. high	1.09 (0.81-1.46)	0.582	1.06 (0.79-1.42)	0.711	1.07 (0.79-1.44)	0.670	1.07 (0.79-1.44)	0.657
	Middle-high vs. high	1.09 (0.80-1.49)	0.567	1.08 (0.79-1.47)	0.629	1.07 (0.79-1.46)	0.658	1.09 (0.80-1.49)	0.585
**Current smoking**	Yes vs. no	0.89 (0.65-1.22)	0.460	0.87 (0.64-1.18)	0.369	0.90 (0.66-1.23)	0.498	0.85 (0.62-1.17)	0.326
**Alcohol consumption**	High-risk drinking vs. abstinence	1.17 (0.80-1.70)	0.418	1.17 (0.80-1.70)	0.422	1.17 (0.81-1.71)	0.406	1.14 (0.78-1.66)	0.500
	Moderate drinking vs. abstinence	1.17 (0.96-1.42)	0.119	1.18 (0.97-1.43)	0.104	1.17 (0.96-1.42)	0.122	1.17 (0.96-1.42)	0.122
**Physical activity**	Active vs. inactive	0.83 (0.65-1.06)	0.140	0.83 (0.65-1.06)	0.142	0.83 (0.65-1.06)	0.143	0.83 (0.65-1.06)	0.146
**Hypertension**	Yes vs. no	2.01 (1.71-2.37)	<0.001	2.03 (1.73-2.40)	<0.001	2.02 (1.71-2.37)	<0.001	2.03 (1.72-2.39)	<0.001
**Diabetes mellitus**	Yes vs. no	1.74 (1.47-2.06)	<0.001	1.75 (1.48-2.07)	<0.001	1.74 (1.47-2.06)	<0.001	1.76 (1.49-2.08)	<0.001

Values are adjusted odds ratios (aORs) with 95% confidence intervals from survey-weighted logistic regression.
Breakfast, lunch, dinner, and the overall regular-meal pattern were analyzed in separate regression models; estimates from the separate models are presented together for comparison.
All Model 3 analyses were adjusted for age, sex, marital status, education level, household income, current smoking, alcohol consumption, physical activity, hypertension, and diabetes mellitus.
Reference outcome category: no obesity. Reference covariate categories are shown in the Comparison column.
†For the breakfast, lunch, and dinner models, regular consumption was defined as 5-7 times/week and compared with ≤4 times/week. For the overall-pattern model, regular consumption of all three meal types was compared with regular consumption of two or fewer meal types.
Abdominal obesity was defined using the Korean criteria (WC ≥90 cm for men and ≥85 cm for women).
Abbreviations: aOR, adjusted odds ratio; CI, confidence interval; WC, waist circumference.

Table S3. Comparison of characteristics between included and excluded participants aged ≥65 years in KNHANES VIII (2019-2021).

Characteristic	Category	Included participants (n = 3,559) n (weighted %)	Excluded participants[a]		*p* value[b]
			Available n	n (weighted %)	
**Sex**	Male	1,558 (44.3)	1,611	658 (41.0)	0.041
	Female	2,001 (55.7)		953 (59.0)	
**Marital status**	Married	2,409 (67.6)	1,605	981 (63.2)	<0.001
	Divorced/separated	217 (6.3)		84 (4.9)	
	Widowed	901 (25.2)		528 (31.3)	
	Never married	32 (0.9)		12 (0.6)	
**Education**	Elementary school or less	1,877 (50.5)	955	520 (51.4)	0.824
	Middle school	623 (17.9)		152 (17.6)	
	High school	682 (20.3)		190 (20.9)	
	College or higher	377 (11.4)		93 (10.1)	
**Household income**	Low (Q1)	1,570 (41.5)	1,563	793 (47.6)	0.010
	Lower-middle (Q2)	1,050 (29.4)		431 (27.8)	
	Upper-middle (Q3)	590 (17.8)		210 (15.6)	
	High (Q4)	349 (11.3)		129 (9.0)	
**Current smoking**	Yes	333 (9.8)	1,491	143 (10.1)	0.814
	No	3,226 (90.2)		1,348 (89.9)	
**Alcohol consumption**	High-risk drinking	178 (5.5)	1,503	63 (4.8)	0.013
	Moderate drinking	1,022 (28.5)		361 (24.2)	
	Non-drinking	2,359 (66.0)		1,079 (71.1)	
**Physical activity**	Active	442 (13.2)	969	95 (10.4)	0.062
	Inactive	3,117 (86.8)		874 (89.6)	
**Hypertension**	Yes	2,229 (62.2)	1,541	978 (63.6)	0.432
	No	1,330 (37.8)		563 (36.4)	
**Diabetes mellitus**	Yes	1,076 (29.7)	1,138	345 (30.6)	0.615
	No	2,483 (70.3)		793 (69.4)	
**Metabolic syndrome**	Yes	1,110 (31.3)	1,313	462 (37.2)	<0.001
	No	2,449 (68.7)		851 (62.8)	
**General obesity**	Yes	1,314 (36.8)	1,458	507 (34.5)	0.207
	No	2,245 (63.2)		951 (65.5)	
**Abdominal obesity**	Yes	1,824 (51.2)	1,577	779 (50.2)	0.541
	No	1,735 (48.8)		798 (49.8)	
**Number of meal types regularly consumed[c]**	≤1	96 (3.0)	921	30 (3.4)	0.835
	2	541 (16.0)		142 (15.8)	
	3	2,922 (81.0)		749 (80.8)	

Values are unweighted counts and survey-weighted percentages. Percentages are calculated within each group using the available observations for each characteristic and may not sum to 100% because of rounding.

[a] Of the 5,285 eligible participants, 1,726 were excluded from the main analysis because of missing data in one or more study variables. Comparisons used the available data for each characteristic; therefore, the number of excluded participants contributing to each comparison varies and may be smaller than the total excluded sample. The variable-specific sample sizes are shown in the table.

[b] p values are based on the adjusted F statistic corresponding to the second-order Rao-Scott correction of the Pearson chi-square test, accounting for the complex survey design.

[c] Regular consumption was defined as consumption 5-7 times per week for each of breakfast, lunch, and dinner. Categories represent the number of meal types regularly consumed, not the actual daily meal count.

KNHANES, Korea National Health and Nutrition Examination Survey; Q, quartile.

Table S4. Sensitivity analysis using the original four meal-frequency categories and Model 3 adjustment among Korean older adults (N = 3559).

Meal	Frequency	Participants n (weighted %)	General obesity			Abdominal obesity		
			Cases n	aOR (95% CI)	*p*	Cases n	aOR (95% CI)	*p*
**Breakfast**	5-7 times/week	3,280 (91.4)	1193	0.61 (0.37-1.02)	0.058	1674	0.77 (0.49-1.22)	0.263
	3-4 times/week	92 (2.8)	46	1.25 (0.66-2.38)	0.488	54	1.31 (0.68-2.52)	0.422
	1-2 times/week	68 (2.1)	24	0.60 (0.28-1.28)	0.184	32	0.67 (0.35-1.29)	0.230
	Almost never	119 (3.7)	51	1.00 (Reference)	-	64	1.00 (Reference)	-
**Lunch**	5-7 times/week	3,246 (90.8)	1184	0.61 (0.41-0.92)	0.017	1645	0.59 (0.38-0.92)	0.019
	3-4 times/week	130 (3.6)	58	0.89 (0.50-1.58)	0.692	77	0.89 (0.48-1.64)	0.708
	1-2 times/week	70 (2.3)	26	0.54 (0.29-0.99)	0.046	39	0.75 (0.37-1.52)	0.431
	Almost never	113 (3.3)	46	1.00 (Reference)	-	63	1.00 (Reference)	-
**Dinner**	5-7 times/week	3,407 (95.5)	1238	0.58 (0.27-1.22)	0.149	1735	0.98 (0.46-2.10)	0.964
	3-4 times/week	84 (2.4)	42	1.38 (0.58-3.28)	0.472	50	1.81 (0.74-4.46)	0.196
	1-2 times/week	35 (1.1)	18	0.80 (0.29-2.25)	0.676	20	0.90 (0.30-2.65)	0.843
	Almost never	33 (1.1)	16	1.00 (Reference)	-	19	1.00 (Reference)	-

Abbreviations: aOR, adjusted odds ratio; BMI, body mass index; CI, confidence interval; KNHANES, Korea National Health and Nutrition Examination Survey.

Participant and case counts are unweighted; percentages are survey-weighted within each meal type and may not sum to 100% because of rounding. The same 3,559 complete-case participants contribute to each meal-specific distribution.

Breakfast, lunch, and dinner were entered separately as categorical predictors in complex-sample logistic regression models. Model 3 adjusted for age, sex, marital status, education, household income, smoking, alcohol consumption, physical activity, hypertension, and diabetes mellitus. Almost never was the reference category for every meal.

General obesity was defined as BMI ≥25 kg/m²; abdominal obesity was defined as waist circumference ≥90 cm in men and ≥85 cm in women. Cases are the number of participants meeting the corresponding outcome definition within each frequency category.

P-values are nominal, two-sided tests of each category versus almost never, without multiplicity adjustment; they are not overall tests of the four-category predictor. Confidence intervals are also unadjusted for multiplicity. These exploratory analyses used a different reference category from the primary dichotomous analyses. The composite indicator of regularly consuming all three meal types was not reclassified.
